# Mediterranean Coastal Lagoons: The Importance of Monitoring in Sediments the Biochemical Composition of Organic Matter

**DOI:** 10.3390/ijerph16183466

**Published:** 2019-09-18

**Authors:** Monia Renzi, Francesca Provenza, Sara Pignattelli, Lucrezia Cilenti, Antonietta Specchiulli, Milva Pepi

**Affiliations:** 1 Bioscience Research Center, Via Aurelia Vecchia, 32, 58015 Orbetello (GR), Italy; francesca.provenza@bsrc.it (F.P.); sara.pignattelli@bsrc.it (S.P.); 2 Department of Lesina (FG), National Research Council—Institute for Biological Resources and Marine Biotechnologies (IRBIM), Via Pola 4, 71010 Lesina, Italy; lucrezia.cilenti@cnr.it (L.C.); antonietta.specchiulli@fg.ismar.cnr.it (A.S.); 3 Stazione Zoologica Anton Dohrn, Villa Comunale, 80121 Napoli, Italy; milva.pepi@szn.it

**Keywords:** decomposition, transitional water ecosystems, organic loads, mesocosm, monitoring programs

## Abstract

Transitional water ecosystems are targeted by the European Union (EU) Water Framework Directive (WFD, CE 2000/60) monitoring programs in coastal zones. Concerning sediments, activities performed for the WFD focus on a few variables concerning the biochemical composition of organic matter. Our research reports the effects of oxygen availability on the biochemical composition of organic matter in sediments to highlight levels of targeted variables in time and, according to the depth of sediment layer, both under oxygenated and anoxic conditions in a mesocosm study on sediment cores. Results provide evidence that tested factors of interest (i.e., disturbance type, oxygenic *versus* anoxic conditions; persistence time of disturbance, 0–14 days; penetration through sedimentary layers, 0–10 cm depth) are able to significantly affect the biochemical composition of organic matter in sediments. Large part of the variables considered in this study (total organic carbon (TOC), total phosphorous (TP), total sulphur (TS), Fe, carbohydrates (CHO), total proteins (PRT), biopolymeric carbon (BPC), chlorophyll-a (Chl-*a*) are significantly affected and correlated to the oxygenation levels and could be good early indicators of important changes of environmental conditions. Monitoring activities performed under WFD guidelines and management strategies of Mediterranean coastal lagoon ecosystems shall include the biochemical composition of organic matter in sediment to provide an exhaustive picture of such dynamic ecosystems.

## 1. Introduction

The Water Framework Directive (WFD, European Union CE 2000/60) defines transitional waters as “bodies of surface water in the vicinity of river mouths which are partly saline in character as a result of their proximity to coastal water, but which are substantially influenced by freshwater flows”. The main physical factors that contribute to the genesis and characterization of coastal lagoons are coastal typology, tidal range, and climate. The former classification of coastal lagoons in transitional waters and coastal waters on the basis of freshwater influence is not suitable for Mediterranean lagoons. Recent literature proposed to make the administrative boundaries consistent with the natural ones by the division in two homogeneous groups of lagoons: Mediterranean lagoons (Mediterranean climate, hot summer, dry season, nanotidal range) and Northern Adriatic lagoons (humid subtropical climate, microtidal range) [1]. Mediterranean lagoons are, in large part, areas of shallow, coastal water, wholly or partially separated from the sea by sandbanks, shingle or, less frequently rocks [2]. Such ecosystems experience fluctuations of environmental variables over ranges of scale and duration, both in space and in time, which are unique and not recorded in other aquatic habitats [3].

Natural drivers as well as climate, rains, and tides, significantly affect oxygen dynamics conditioning the occurrence of water eutrophication in Mediterranean lagoons [4]. Eutrophication is a well-known phenomenon supported by nutrient enrichments that cause a significant increase of primary productivity, which could determine a notable reduction of the secondary ones [5]. The introduction of the WFD notably increased the knowledge on coastal lagoons in Europe. Mediterranean lagoons are widely affected by water eutrophication [6]. Under eutrophic conditionsdynamics occurring in abiotic matrices and effects on biological populations are widelydocumented by the literature [2]. Mediterranean coastal wetlandsare highly affected by agricultural activities, and external freshwater inputs are considered the main driver of nutrient supplies (mainly as inorganic nitrogen) causing a fertilization effect [7,8,9]. Furthermore, domestic, and industrial effluents discharge a high load of nutrients and organic inputs in the lagoon from the surrounding areas [10].

For the exposed reasons, Mediterranean lagoons evidence a wide variability of physical-chemical drivers in water as reported by literature [11]. Principal descriptors of water trophic levels, such as N-NH_4_^+^ (<limit of detection (LOD)-30.9 μM [4,12,13]), N-NO_2_^−^ (<LOD-25.9 μM [12,13]), N-NO_3_^−^ (<LOD-156 μM [14,15]), SRP (<LOD-10.2 μM [11]), and chlorophyll-a (<LOD-56.3 μg/L [16]), ranged widely seasonally.

The ecological status is addressed by the WFD to preserve and manage, in a sustainable way, aquatic ecosystems. Even if the integration of assessments based on a considered element for the ecosystem quality is a key step for the ecological status assessment of lagoon ecosystems it was not developed by WFD [17]. In fact, WFD proposed procedures based on a single biological quality element [18]; the assessment of functional properties of the whole ecosystem is lacking. Furthermore, responses to stress are still almost completely lacking, mainly for transitional water ecosystems [19]. In fact, *sensu* WFD, the ecological status is measured at the level of the main guilds (i.e., biological quality elements, BQEs). By contrast, from an ecological point of view, ecosystem health is more properly measured on the basis of complex ecosystem properties (i.e., vigour, organisation, and resilience) *sensu* Costanza [20] as reported by literature [19].

Concerning abiotic drivers, in such dynamic and fluctuating ecosystems, the WFD includes the monitoring of a small group of physical-chemical variables in water (i.e., dissolved inorganic nitrogen, orthophosphates, oxygen), and sediments. The larger part of monitoring programs is focused on biota and physical-chemical variables are considered only as a general frame and supporting data for the biotic-based classifications. In spite of that, several factors, such as geomorphology, morphometry, hydrology, hydrodynamics, climatic conditions, and nutrient loads, are identified to drive/modulate chemical reactions and metabolic pathways affecting biota [10]. Furthermore, humans can manage some of the drivers considered able to affect trophic level in lagoons (i.e., water exchanges, bottom depth, human effluents, and oxygen availability) by forcing significantly the ecosystem health. It was reported by literature [19] that during a dystrophic event that occurred in Lesina Lagoon (Italy), some variables (i.e., O_2_, NH_4_^+^, TP, Chl-*a*, in water; redox potential, TN, TP in sediment) showed earlier alterations during dystrophic crises. Authors evidenced as monitoring strategies should incorporate early warning descriptors useful to evaluate and describe temporal variation of the ecosystem resistance and, also, evidenced that non-taxonomic ones should address descriptor-specific typology [21], uncertainty [22], and type-specific conditions [21].

The starting point of macroalgae proliferation is when the natural balance is disturbed by natural drivers (light availability, rains, wind, etc.) but, also, by nutrient release towards the water column from stocks stored in sediments [10]. The decomposition of organic matter in such ecosystems is controlled by a complex array of processes, including sedimentation, transformation, accumulation and export, which affect the content and chemical composition of the organic molecules [2].

Concerning chemical composition of organic matter in sediments, protein level is associated to the productivity of a given marine ecosystem [23]. In fact, different trophic conditions could determine significant changes on both quantity and quality in term of biochemical composition of organic matter in sediment. Other authors [24], evidenced that total organic matter, chlorophyll-a, phaeopigment, protein, lipid, carbohydrate, and biopolymeric carbon concentrations in sediments are good proxies of the trophic level of lagoon ecosystems. Furthermore, authors founded that exploring levels of phyto-pigments in sediments resulted in being a powerful tool, more informative than chlorophyll-a in water, to determine the occurrence of different types of anthropogenic pressures.In cited research, authors evidenced that the largest part of coastal lagoons that resultedin being oligotrophic according to a classification based on physical-chemical descriptors of water, such aslevels of inorganic nutrient and chlorophyll-a, resulted in being far more eutrophic than expected when classification were performed focusing on organic matter in sediments. 

In spite of the importance of sedimentary organic matter, nevertheless, quantitative and qualitative relationships among oxygen availability at the sedimentary level and changes of chemical composition of the stored organic matter are not yet completely clarified by the literature. Some researches performed in field, evidenced that resuspension of bottom sediments could modify the chemical composition of the organic matter of settling particles both decreasing the labile fraction of and increasing the more refractory fraction of the organic matter [25,26]. Nevertheless, field research suffersfrom large interferences driven by natural factors (i.e., water temperature, irradiation, wind, currents, etc.) that affectobserved variance of chemical composition of organic matter in sediments and that are difficult to isolate. For these reasons, dynamicsaffecting the organic matter composition in sediments related to the oxygen availability should be studied also under standardized conditions to better understand complex phenomena occurring under natural conditions. 

The aim of this study is to contribute to a major understanding onthe biochemical composition of organic matter and decomposition processes occurring in sediments under oxygenation and anoxic conditions induced artificially. Compared to research performed in the field, our research, developed under laboratory-controlled conditionsallows us to better standardize some external factors of variability among tested treatments such as:chemical composition of the sedimentary organic matter at the starting time;the amount of oxygen/nitrogen fluxed during the experiment; other external factors affecting natural fluctuations (i.e., air temperature, light/dark cycles, winds, animal and human interferences, etc.). Furthermore, observed effects are related also to the duration of the treatment and to the depth layer of sediment. Obtained results will allow us to select specific variables that could earlier evidence ecosystem changes to the development of larger monitoring programs in lagoon ecosystems in order to early detect ecosystem evolution towards eutrophication or the occurrence of a dystrophic crises. 

## 2. Material and Methods

### 2.1. Sampling Area: The Orbetello Lagoon

**Figure 1:**
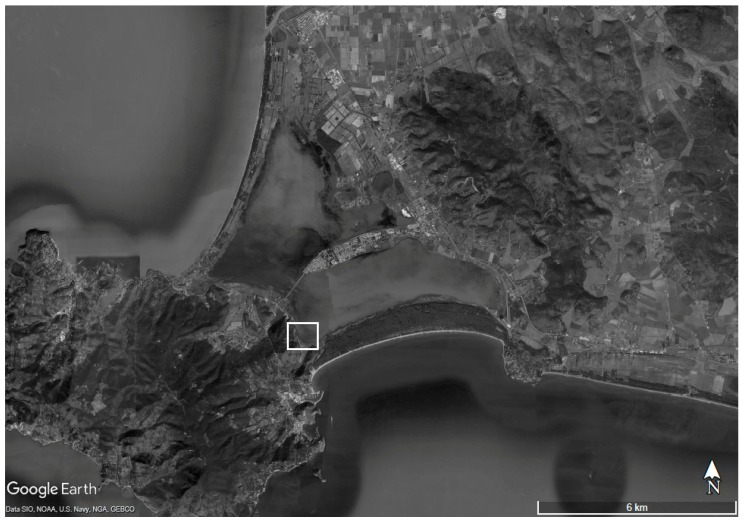
Study area (Orbetello Lagoon) and sampling area selected for sediment cores collection (whitesquare).

The study areawas selected in the Orbetello Lagoon (Central Tyrrhenian Sea, Italy) on the basis of a solid long-term physical-chemical database on water and sediment characteristics collected on this lagoon ecosystem that allow us to localize well-delineated areas that better fit with the aim of this research. Starting variability among collected sediment cores, low-scale geographic variability of the organic matter composition, and other factors that could affect the biochemical composition of organic matter and, consequently, tested performances were reduced by selecting a homogeneous sampling area within the lagoon on the basis of previous research [4,27,28,29]. Sediment cores were collected in the pre-selected and homogeneous area of the Orbetello Lagoon (42°25′–42°29′ North; 11°10′–11°17′ East) represented in Figure 1. Selected sampling area was characterized by a low depth of water (<30 cm [30]); low water renewal [30]; high content of silt in sediments (>90% of particles lower than 63 μm [31]); very low redox potential in sediments (lower than −400 mV [30]); the absence of rooted vegetation and seasonal coverage by *Chaetomorpha linum* [32]; and homogeneous levels of total organic carbon (TOC) [31].

### 2.2. Logic Model and Experimental Conditions

#### 2.2.1. Logic Model

Experiments were sized to reduce Type-I and Type-II errors according to a logical model [33,34,35] based on a nested hierarchical design developed according to four *a priori* randomly defined sediment factors. The disturbance type (DT) had two levels fixed: oxygenic conditions (H), and anoxic conditions (A). The persistence time of disturbance (PTD) showed four temporal fixed levels: controls, starting time (T_0_), three days (T_1_), seven days (T_2_), and 14 days (T_3_) after starting the treatment. The penetration through sedimentary layers (PTSL) showed five fixed levels (0–2, 2–4, 4–6, 6–8, 8–10 cm). Replicates were performed on three random levels. A total of 120 records were collected on the sediment (*n* = 120).

#### 2.2.2. Sample Collection and Experimental Conditions

Sediment cores were collected through the direct insertion in the sediment for almost 30 cm of depth of a polycarbonate core tube (50 cm length, 10 cm diameter). All the sediments cores needed to perform the whole experiment were collected at the same time and closed together from a homogeneous area as previously described to reduce natural micro-scale variability of the bottom and to minimize significant differences of starting condition among tested sediment cores. Collected cores were completely filled by about 20–25 cm of sediments and 25 cm of column water. Cores embedded in the bottom were sealed using a Teflon plug before being removed from the bottom. Sealed collected cores were carried under vertical and refrigerated conditions to the laboratory to reduce disturbance. Incubation experimentsstarted immediately after sampling under controlled conditions. Three sediment cores per treatment (*n* = 6), randomly extracted among the whole amount of sediment cores collected (*n*=24) were used to determine sediment control conditions at each tested PTSL and assuming recorded levels as starting values (T_0_). Room temperature was fixed at 25 °C for the whole experiment on the basis of the mean spring-summer temperature of the largest part of the Mediterranean lagoons. The light/dark cycles were set 12:12 by exposure to artificial white light (6000 lux). Anoxic and oxygenated conditions were obtained respectively by fluxing in water and core headspace respectively nitrogen (N_2_) and oxygen (O_2_) ultrapure gasesto create artificially stable and persistent conditions.The flow system used was hermetically sealed to the caps in the upper part of the cores in order to guarantee anoxic / oxygenated conditions.In both cases, an opportune flux reduction system was assembled to perform fluxing without excessive water turbulences. Sol spa (Italy) purchased analytical grade gases used during our experiments. Levels of DO in water were checked, ensuring anoxia, before the hermetical closure of the whole group of “A” cores (DO = 0%). The oxygen was directly fluxed within the water layer by a pressure-reduced oxygen tank. The oxygenation levels in “H” treated cores were daily checked in the water layer by a Hanna field probe to ensure oversaturation (DO > 100%). At each experimental time (PTD) a group of three both “A” and “H” treated cores were randomly drawn and sampled to perform sediment analyses. The experiment lasted a total of two weeks. During this time, samples were taken under control conditions (T_0_) and after 3 days (T_1_), 7 days (T_2_) and 14 days (T_3_) of treatment.

#### 2.2.3. Sample Pre-Treatments

The water overlaying sediments were used to convey the treatment gas and to measure O_2_ levels ensuring the maintenance over time of the desired experimental conditions (oversaturation for O_2_ and anoxic condition for N_2_ fluxed cores. Sediment cores (0–10 cm) were extracted from the liner onto a rinsed aluminium foil and sectioned at depth layers of 2 cm of thickness to obtain five sub-samples sediment from each sediment core for the evaluation of chemical levels at each PTSL. To reduce interference during sampling, anoxic treatedcores were collected under anoxic conditions. Under a continuous N_2_ gas flux, anoxic treated cores were extracted from the core tubes to reduce air exposure. Extracted sediments were subsampled immediately and stored in N_2_ fluxed bottles without headspace. Recovered sediments measured about 20–25 cm of length but only 15 cm of them were analysed at the end of the exposure experiment. In fact, about 5 cm of the sediment layer settled at the end of the core tube and contacting the core plug (Teflon) was thrown away to avoid interference. All the collected samples were stored at −20 °C and analysed within few days. Before analysis, sediment samples were lyophilized and accurately homogenized to reduce the matrix variability. Large components, if present, were removed mechanically by sieving using a >1 mm Ø steel test sieve DIN EN ISO, 9001.

### 2.3. Laboratory Analyses

Each sediment sample was analysedto determine chlorophyll-*a* (Chl-*a*), phaeopigments (PHEO), total proteins (PRT), carbohydrates (CHO), total lipids (LIP), labile organic matter (LOM), refractory organic matter (ROM), total organic matter (OM), total organic carbon (TOC), total carbon (TC), total nitrogen (TN), total sulphur (TS), total phosphorous (TP), and total iron (Fe) levels.

Chl-*a* and PHEO pigments (µg/g) were extracted in 90% acetone and spectrophotometrically measured before and after acidification with HCl 1N, according to the methodology reported in literature [36]. The total phaeopigment concentrations resulted from the sum of Chl-*a* and PHEO [24]. The colorimetric method [37] was followed for PRT determination (mg/g), using a standard solution of albumin (Sigma Aldrich); protein concentrations were expressed as bovine serum albumin equivalents. The methodology reported in [38], optimized for sediments by Gerchakov and Hatcher [39] was used for spectrophotometric determination of CHO (mg/g), after instrument calibration with a standard solutions of D(+)-glucose. Total lipids (mg/g) were determined by the spectrophotometric method [40], after extraction with a mixture of methanol and chloroform (1:1 v/v) according to Bligh and Dyer [41]; were expressed in tripalmitin equivalents.

The correction factors in PRT, CHO and LIP were determined on sediment replicates (5 replicates), according to literature [42]. Biopolymeric carbon (BPC, mgC/g) was computed as the sum of carbon equivalents of CHO, PRT and LIP, according to literature [43].

LOM and ROM were measured by loss of weight after combustion for 3 h at 250 °C and 400 °C respectively, while OM was calculated as sum of the two fractions [44,45]. LOM, ROM and OM values were expressed as %. Total organic carbon (TOC), total carbon (TC), total nitrogen (TN) and total sulphur (TS) contents were determined using an elemental analyser (CHNS/O 2400, Perkin Elmer) and expressed as % d.w. Total phosphorous (TP, %; [46]) and total iron (Fe, mg/kg; [47]) were determined by spectrophotometric methods using a 6505 UV/Vis (Jenway) dual beam spectrophotometer after acid digestion of sediment samples.

Recoveries and reproducibility were checked by analysing procedural blanks and performing laboratory inter-calibrations on similar matrices. Standard reference solutions for each measured variable, were analysed in statistical replicates (*n* = 10) to calculate averages and standard deviation (SD) of recoveries which ranged from 0.2–0.5%. Average percentages of recoveries were within 92–102.3% range of variation and analytical concentrations were not recovery corrected. Limit of detection (LOD) was defined as the average blank (*n* = 10) plus three standard deviation (SD); limit of quantification (LOQ) for the adopted procedures were calculated by progressive serial dilution of standard solution of 0.1 µM for all determined nutrients.

### 2.4. Statistical Analysis

Univariate and multivariate analyses were performed with GraphPad Prism v.5.0 (GraphPad Software, San Diego California USA, www.graphpad.com) and Primer v.6.0 (Primer-E Ltd., Plymouth Marine Laboratory, UK) packages. Analysis of variance (ANOVA) tests were performed to evaluate significance according to tested factors of interest: disturbance type (DT), persistence time of disturbance (PTD), and penetration through sedimentary layers (PTSL).

Relationships among variables were observed by the Pearson’s correlation matrix (*p* < 0.01) and uncorrelated variables were selected in order to perform multivariate routines. Principal component analysis (PCA) and non-metric multi-dimensional scaling (nm-MDS) were performed on pre-treated data by the application of square root and log(x + 1) to test the significance level of observed segregations according to *a priori* defined factors. Observed dissimilarities between groups were also tested by the ANOSIM (ANnalysis Of SIMilarities) R statistic test, using permutation/randomization methods on a resemblance matrix and performing 9999 runs [48].

## 3. Results

### 3.1. Macroelements

Table 1 and Table 2**,** reports mean chemical composition of total carbon (TC), total organic carbon (TOC), total nitrogen (TN), total sulphur (TS), total phosphorus (TP), iron (Fe) and their associated standard errors respectively under exposure to oxygenic (H) and anoxic (A) conditions. Data are grouped for each tested deep layer (PTSL, *n* = 5) at each tested treatment time (PTD, *n* = 4), results are compared to control conditions (T_0_ column).

Under both tested condition, total carbon (TC) levels appear significant in three PTSL and the trend is not regular. Overall, under anoxic condition (Table 2), TC mean values are higher than oxygenic. Under oxygenic condition, levels of total organic carbon (TOC) are always significantly different concerning levels (Table 1). Trends from T_0_ to T_1_ are opposite to trends recorded from T_1_ to T_2_. In particular, concerning 0–2, 2–4, and 8–10 cm depths, means show an increase, then a decrease; while for 4–6 cm, and 6–8 cm depths, an opposite behaviour was recorded. Under anoxic condition (Table 2), the factor penetration through sedimentary layers (PTSL) resulted always significant with the exception of 8–10 cm. Compared to oxygenic condition, trends result different only for 0–2 cm and 8–10 cm depths.

Under oxygenic condition, total nitrogen (TN) recorded statistically significant for all PTSL except for 8–10 cm; at each level it increases from T_0_ to T_1_ and then decrease in T_2_ and T_3_ (Table 1). Under anoxic conditions (Table 2), the only significant level is 2–4 cm, which increases just from T_0_. Total sulphur (TS) content, under both oxygenic (Table 1) and anoxic (Table 2) condition; shows all significant levels with the exception of 0–2 cm under oxygenic conditions. It should be noted that the major increasing from T0 to the end of the experiment is recorded in 0–2 cm depth under anoxic conditions and in 8–10 cm depth under oxygenic conditions, literally the opposite behaviour.

Under oxygenic condition (Table 1), total phosphorus (TP) shows 0–2 cm and 8–10 cm levels as statistically significant. Interestingly, this element shows lower values than other elements and the first level shows an increasing trend from T0, conversely 8–10 cm level has an opposite trend. Instead, under anoxic condition (Table 2), TP shows that the first three levels are statistically significant.

Under oxygenic conditions (Table 1), iron mean concentration (Fe) is always significant for each level. Under anoxic conditions (Table 2), the only not significant level is 4–6 cm. Under this condition, from T_0_ to T_3_, the value always increases (except in 6–8 cm depth); instead, under oxygenic condition, the value decreases most frequently (in three levels, from 2 to 8 cm depth). Interestingly, under both DL (oxygenic and anoxic conditions) T_2_ and T_3_ show always the same values.

### 3.2. Organic Matter Composition

In Table 3, mean and standard error levels of carbohydrates (CHO), proteins (PRT) and lipids (LIP) composition are reported for each deep layer (PTSL) in each time (PTD) under oxygenic conditions. In Table 4, the same data are reported under anoxic conditions.

Carbohydrates composition (CHO) under oxygenic (Table 3) and anoxic (Table 4) conditions shows irregular trend, but the last two levels (6–8 cm and 8–10 cm) show the same value.

**Figure 2:**
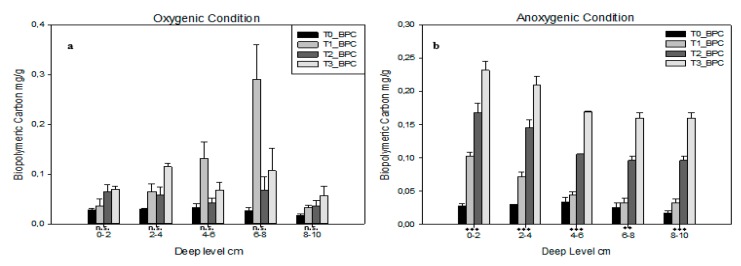
Biopolymeric carbon (BPC) for each deep level in each time in (**a**) oxygenic and (**b**) anoxic conditions. Error bars indicate one standard error either side of the mean. P-level of ANOVA is indicated (*, *p* < 0.05; **, *p* < 0.01; ***, *p* < 0.001; ns, not significant).

Concerning proteins’ composition (PRT) it is interesting to underline that, under both conditions, the trend increases, but in anoxic conditions (Table 4) it increasessignificantly. In fact, *p*-level is not significant under oxygenic conditions (Table 3) and significant with *p* < 0.01 under anoxic conditions. Lipids composition (LIP) results statistically significant only in 4–6 cm and 6–8 cm levelsunder oxygenic conditions (Table 3). Biopolymeric carbon (BPC) content is shown in Figure 2; under oxygenic conditions (Figure 2a) none of the levels analysed are statistically significant. The higher concentration of BPC is shown by T1 at 6–8 cm depth; here T_1_ shows an increasing trend from the most superficial layer to 6–8 a then a decreasing trend. In both the first and the last depth layers, BPC concentration are almost the same for each PTD. Under anoxic conditions (Figure 2b), BPC shows a completely different behaviour: all layers are statistically significant. In each level, BPC content shows an increasing trend from T_0_ to T_3_. Under oxygenic conditions, all T_1_, T_2_ and T_3_ show a decreasing trend from 0–2 to 8–10 layer.

**Figure 3:**
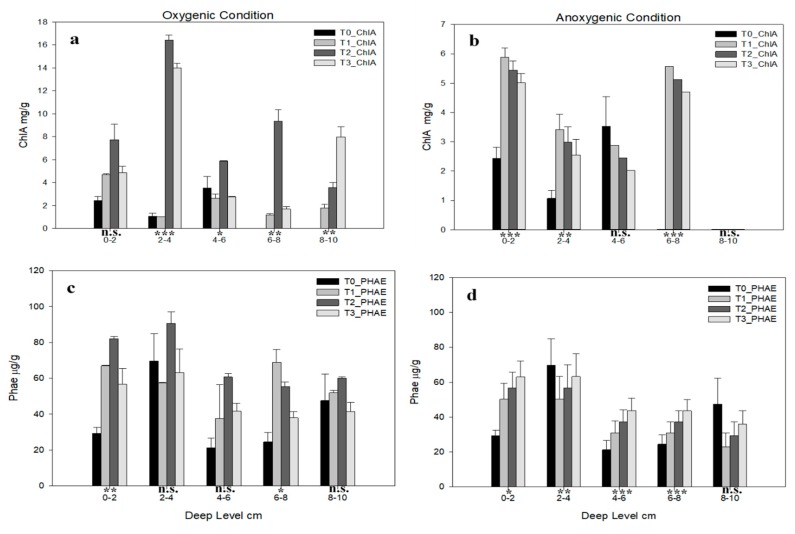
Pigments concentration for each deep level in each time in (**a**) chlorophyll-A (ChlA) oxygenic and (**b**) anoxic conditions, (**c**) pheophytins (Phae) oxygenic and (**d**) anoxic conditions. Error bars indicate one standard error either side of the mean. P-level of ANOVA are indicated (*, *p* < 0.05; **, *p* < 0.01; ***, *p* < 0.001; ns, not significant).

Pigments content are shown in Figure 3. Under oxygenic condition (Figure 3a), Chlorophyll-*a* (Chl-*a*) is statistically significant in each level except in 0–2 cm. Under anoxic condition (Figure 3b) the only not statistically significant level is 4–6, and Chl-*a* content is always lower than oxygenic condition. Interestingly, at the levels 0–2 cm, 2–4 cm and 6–8 cm, graphic shows a decreasing trend from T_1_ to T_3_. In both graphs this pigment has a discontinuous pattern along the sampling depths.

The phaeophytin contents (PHAE) in oxygenic conditions are shown in Figure 3c, here the only levels statistically significant are 0–2 cm and 6–8 cm depth. In 0–2 cm depth the highest concentration is shown to T_2_ followed by T_1_ and T_3_, while in 6–8 cm there is an opposite trend. In anoxic conditions (Figure 3d), the only level not statistically significant is 8–10 cm; all the other levels show an increasing trend from T_0_ to T_3_.

**Figure 4:**
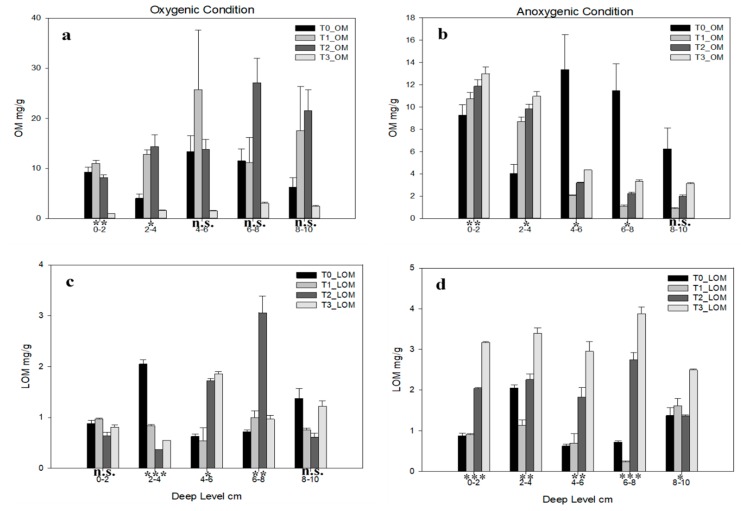
Organic matter concentration for each deep level in each time in (**a**) total organic matter (OM) in oxygenic and (**b**) anoxic condition, (**c**) labile organic matter (LOM) in oxygenic and (**d**) anoxic conditions. Error bars indicate one standard error either side of the mean. *p*-level of ANOVA are indicated (*, *p*< 0.05; **, *p*< 0.01; ***, *p*< 0.001; ns, not significant).

Different kind of organic matters are shown in Figure 4; in oxygenic conditions, total organic matter (OM) (Figure 4a) is statistically significant only in 0–2 cm and 2–4 cm levels; T1 shows an increasing trend until 4–6 cm, whereas higher T2 OM values are shown in the last two depths. In anoxic conditions (Figure 4b) the only not statistically significant level is 8–10 cm. All the levels show an increasing trend from T_1_ to T_3_, but in 0–2 cm, 2–4 cm, and 4–6 cm depth levels the OM content is higher in T_0_ than in other time.

Labile organic matter (LOM) content (Figure 4c,d) is always lower than OM; in oxygenic conditions the only statistically significant levels are 2–4 cm, 4–6 cm, and 6–8 cm depth, in this layer T2 shows the same behaviour of total organic matter. The anoxic condition shows all statistically significant differences among levels, for each level higher content is shown by T_3_ followed to T_2_ mostly; lower concentration in 0–2 cm, 4–6 cm, and 8–10 cm are shown by T_0_.

### 3.3. Multivariate Analyses

An ANOSIM test performed on the whole collected data evidenced that DT conditions produced significant differences concerning biochemical composition of organic matter (Global R: 0.185; significance level of sample statistic: 0.2%; number of permuted statistics greater than or equal to Global R: 19). As data resulted significantly different according to DT, multivariate statistics were performed on separate databases according to DT conditions.

**Figure 5:**
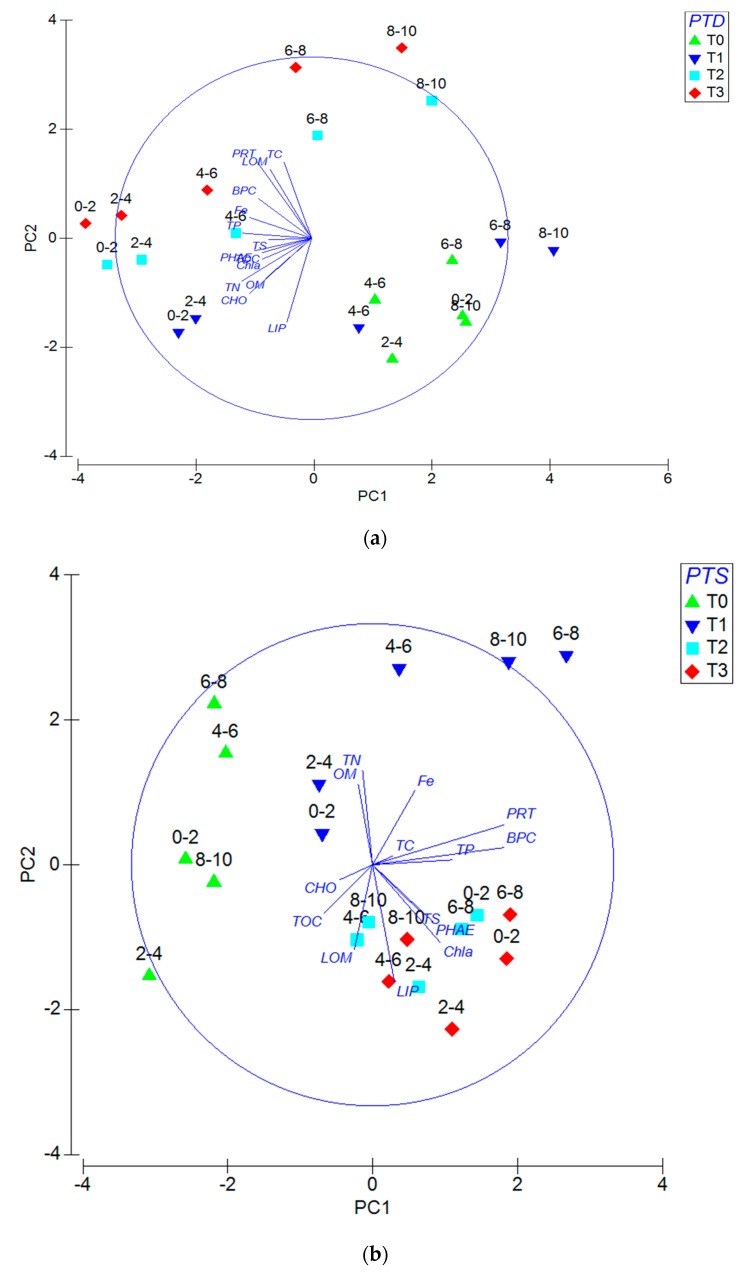
(**a**) Principal component analysis (PCA) performed under oxygenic conditions highlighting the effects according to persistence time of disturbance (PTD) and penetration through sedimentary layers (PTSL) factors. (**b**) PCA performed under anoxic conditions highlighting the effects according to PTD and PTSL factors.

Under oxygenic conditions (Figure 5a), PCA highlights that the first three axes accounted for the 56.4% of the total variance (respectively of 28.1%; 16.4%; 11.9%). Eigenvectors (coefficients in the linear combinations of variables making up principal components) are reported in Table 5a. Under anoxic conditions (Figure 5b), PCA highlights that the first three axes accounted for the 73.6% of the total variance (respectively of 43.3%; 19.6%; 10.7%). Eigenvectors (coefficients in the linear combinations of variables making up PC’s) are reported in Table 5b. Under anoxic condition, ANOSIM test evidence that samples are significantly different concerning the factor PTD (Global R: 0.322; significance level of sample statistic: 0.8%; number of permuted statistics greater than or equal to Global R: 81). Under oxygenated conditions, higher statistically significance of the ANOSIM test concerning the factor PTD is reported (Global R: 0.473; significance level of sample statistic: 0.03%; number of permuted statistics greater than or equal to Global R: 3). The pairwise test performed evidences that under anoxic conditions, principal differences among sediments are recorded between T_0_-T_2_ (significance of 0.8%), while under oxygenic conditions only the T_2_-T_3_ couple shows not significant (5.6%) and all the other tested couples report significant levels (<0.8%). Concerning the tested factor PTSL, the ANOSIM test evidences low significance under anoxic conditions (Global R: 0.216; significance level of sample statistic: 3%) and any significance under oxygenic conditions (Global R: 0.119; significance level of sample statistic: 12.1%). The pairwise test highlights a significant difference under A conditions from 0–2 cm till 4–6 cm (*p* < 5.7%) and under H conditions from 0–2 cm till 2–4 cm (*p* < 2.9%).

## 4. Discussion

Coastal lagoons are human-stressed ecosystems [27,49]) characterized by water eutrophication [4] and by a critical equilibrium among macroalgae and phanerogams habitats [31,32]. In fact, many of such eutrophic coastal lagoons and estuaries produce excessive macroalgal biomass during warmer months [50,51]. In such ecosystem, uneven accumulations of decomposing biomass [30] are frequent and ecosystem could quickly evolve towards a critical point [19]. Numerous reciprocal interfering physical–chemical dynamics regulate nutrient bioavailability affecting the overall water trophic level and, consequently, macroalgae proliferations. Rivers and freshwater inputs drive natural inputs of nutrients and pollutants [4,52,53].

Biomasses exert a key-modulating role on dynamics affecting water and sediment chemistry inducing rapid oxygen/anoxia shifts. A key bottom-up control for the water column quality is represented by the metabolic processes occurring in sediments and involving the release in the water column of organic matter stored in sediments. In coastal lagoons, sulphate-reducing bacteria activelydrive mineralization processes in sediments; it is documented that, in such ecosystems, more than 50% of the sedimentary organic matter is degraded by this bacterial metabolic pathway producing a process named sulphate respiration [54].High contents of sedimentary organic matterimprove decomposition rates by this process and produce toxic gases as by-products such as CO_2_ and hydrogen sulphide (H_2_S). CO_2_ acts to decrease pH in sediments, while H_2_S, that is the predominant sulphur form under highly reducing conditions, is acutely toxic for the biota [55]. The combined effects of these gases on biota is direct in terms of acute toxicity but also indirect in terms of oxygen depletion andcorrelated phenomenaproducing significant ecological and economic consequences on the whole ecosystem. Regarding indirect effects, when oxygen depletion occurs, pH values in sediments shift towards acidity, and redox-potential reduced lower than −400 mV leading to a build-up of both reduced and reducing components. These conditions induce ammonium production [56], nitrite increase by ammonification of organic matter with a toxic effect on biota [57], and an evolution towards the dystrophyimpacting on local economy based on aquaculture, fishing activities, and tourism [4]. Biochemical components of organic matter are strongly modulated by oxygenation levels in sediment layers and by biogeochemical cycles of trace elements. The liberation of free sulphide from sediments is hindered by the presence of ferrous and ferric ions, a natural buffering system, which oxide and blocked H_2_S producing ferrous sulphide (FeS) and then as pyrite (FeS_2_) [58,59,60,61,62]. For this reason, if the organic matter load in sediment does not exceed the iron pool availability (H_2_S/Fe > 1), this natural buffer can block dystrophy [63]. Orthophosphates, previously bound to ferric oxides-hydroxides, are released in a water column if the iron reduction buffer operates [62,64,65] to stimulate macroalgal productivity. Literature evidenced that when oxygen is available, orthophosphates are strongly bound to ferric oxides-hydroxides [65,66] and to carbonates and clays [67,68] and are not released in the water column. Oxidation accelerates the nitrification processes producing nitrate predominance against the presence of reduced forms represented by nitrite and ammonium [69]. On the contrary, anoxic conditions produce high denitrification rates [70] increasing nitrate release from sediments as N_2_ or N_2_O.

The persistence of anoxic conditions in coastal lagoons and the metabolic activation of bacteria associated with the sulphur cycle could mobilize chemical contaminants present in sediments in their mineral form. This is the case of mercury from cinnabar that could be released as methylmercury [71,72]. These processes are strictly linked to biomass proliferation. Literature [73] evidenced as the presence of macroalgae in sediments resulted in a source of labile organic matter able to promote microenvironments favourable for the activity of sulphate-reducing bacteria and methylmercury production. In a lagoon ecosystem, sediments represent a source of contamination by methylmercury for both water and biota [74,75,76]. Total mercury and methylmercury were quantified in several specimens of fish of human interest [77]. Relationships among organic matter composition in sediments and methylmercury production in high reductive ecosystems are reported by the literature, supporting the need to monitor a wider range of variables in sediments. In fact, both the origin and the molecular composition of organic matter are key aspects to understand and predict methylmercury formation and accumulation [78]. Recent research evidenced as, in brackish water, that methylmercury production is stimulated by macroalgae [79,80]. Literature evidenced that labile organic matter originated from algal biomasses are associated to reductive phenomena occurring at the sediment-water interface enhancing methylmercury production [78]. By contrast, rooted vegetation actively oxygenates superficial sediment layers by spreading the oxygen produced by photosynthesis from the rhizosphere [31]. Oxygenation of sediments by rooted vegetation exert a sort of bottom-up control on the quality of organic matter affecting its biochemical composition, on mercury methylation rates, and on the release of chemical substances stored in sediments, producing significant effects on ecosystem health.

A complete monitoring of sedimentary biochemical composition of organic matter could be useful to better understand on-going processes and to quickly prevent possible evolution towards dystrophic crises of such ecosystems. Energy-rich compounds (i.e., peptides, carbohydrates, lipids) that are present in the labile fraction of organic matter are essential macromolecules for microbial metabolic pathways. These compounds show a different composition of levels, forms, and relative proportions in natural environments [24,25], but nevertheless resulted in a better proxy of trophic level than water descriptors [24]. It was reported in the literature that there was a significant increase of the refractory fraction with important changes in its biochemical composition in sediments following short-term storm- and trawling- induced resuspensions [25]. Another research performed in field inducing artificial resuspension of sediments evidenced that labile fraction of the sedimentary organic matter is reducedwith the increase of the refractory fraction [26]. Based on these results, some experiments on artificial disturbance of superficial sediments in coastal lagoons were performed and proposed as a possible solution to manage these environments and to prevent dystrophic crisis [81,82]. Even if artificial sediment resuspension [83] appeared to be an interesting tool to reduce management costs due to classical management approaches (i.e., harvesting and disposal of macroalgae, artificial water exchanges by pumping, sediment dredging operations), nevertheless, specific risks related to the release of chemicals from oxidised sediments [84] shall be accurately explored.

A clear difference between anoxic and oxygenic pathways in the chemical composition of sedimentary organic matter is confirmed by this study. This result is supported by the literature which reported significant and early changes on chemical composition following induced oxygenation of sediments by artificial or natural due disturbances [25,26]. In particular, in oxygenated samples, a significant increase of refractory organic matter associated to a reduction of labile organic matter and protein/carbohydrate ratio is confirmed in this study. Concerning trace elements, oxygenation induces iron oxidation into its ferric forms, which adsorb orthophosphates removing them from the water column and an increase of TP levels in sediments is recorded in time during oxygenation treatments. Anoxic sediments leading to a build-up of both reduced and reducing components.

Results reported by this study evidenced that a large part of variables considered (TOC, TP, TS, Fe, CHO, PRT, BPC, Chl-*a*) are significantly affected and correlated to the oxygenation levels without sediment resuspension processes and could be good early indicators of important changes of environmental conditions. These results enhance the evidence reported by the cited literature that the biochemical composition is a useful tool to monitor ecological status of coastal lagoon ecosystems [24,25,26,81,82]. Furthermore, as reported below, chemical pathways that could be activated in sediments to face phosphorous, ammonium, or sulphated disbalance are complex and multiple interactions occur. For this reason, the level of information acquired on the ecosystem status on the basis of a few variables is lacking and not realistic. Furthermore, another consequence, is that trends recorded for each chemical compound considered in this study during different DT, PTD, and PTSL monitoringare difficult to discuss by considering each variable by itself. This fact, in our opinion, is due to the difficult to understand a multivariable reality on the basis of a univariate point of view. For this reason, the multivariate statistical approach performed in this study allows us to obtain a complete picture of the whole results among multi-layers interactions occurring among variables.

Our results provide evidence that in both oxygenated and anoxic treated cores, chemical composition of the sedimentary organic matter is significantly affected by treatments compared to controls. The penetration thought sediment layers of the effects induced by gas exposure is different comparing treatments. Our results evidence that oxygenation exposure is able to affect biochemical composition of organic matter until 4 cm of sediment depth while anoxic conditions induced changes until 6 cm of depth. In spite of this observed difference, other than the superficial layer of lagoon sediments gas penetration and associated reactions are not able to affect biochemical composition of organic matter in sediments without mechanical or biological sediment disturbance. Finally, our results evidenced that 7 days of exposure are sufficient to significantly change the biochemical composition of organic matter in sediments. These data are of particular relevance to develop predictive models on dystrophic occurrence in coastal lagoon ecosystems.

## 5. Conclusions

This study provides evidence that biogeochemical composition of organic matter in sediment from coastal lagoons is strongly affected by oxygenation levels. Measured variables evidence early fluctuations (7 days of exposure) in superficial layers (<4 cm depth) following oxygen availability and could represent useful indicators of the evolution of environmental conditions. Monitoring activities performed under WFD guidelines and management strategies of Mediterranean coastal lagoon ecosystems shall include biochemical composition of organic matter in sediment to take an exhaustive picture of such dynamic ecosystems.

## Figures and Tables

**Table 1:** Sediments chemical composition for each deep layer in each time under oxygenic conditions.

Content	Depth	T_0_		T_1_		T_2_		T_3_		
**TOC (%)**	cm	Oxy Condition		Oxy Condition		Oxy Condition		Oxy Condition		*p*-level
		mean	(se)	mean	(se)	mean	(se)	mean	(se)	
	0–2	0.580	(0.030)	1.290	(0.020)	0.665	(0.015)	0.665	(0.015)	***
	2–4	0.710	(0.010)	1.295	(0.025)	0.840	(0.030)	0.840	(0.030)	***
	4–6	1.000	(0.010)	0.425	(0.065)	0.745	(0.005)	0.745	(0.005)	**
	6–8	0.615	(0.065)	0.300	(0.010)	0.535	(0.025)	0.535	(0.025)	*
	8–10	0.405	(0.015)	0.305	(0.025)	0.530	(0.040)	0.530	(0.040)	*
**TN (%)**										
	0–2	0.040	(0.010)	0.405	(0.085)	0.030	(0.000)	0.030	(0.000)	*
	2–4	0.215	(0.065)	0.435	(0.065)	0.030	(0.000)	0.030	(0.000)	*
	4–6	0.220	(0.030)	0.530	(0.120)	0.025	(0.005)	0.025	(0.005)	*
	6–8	0.100	(0.050)	0.280	(0.000)	0.075	(0.005)	0.075	(0.005)	*
	8–10	0.080	(0.010)	0.365	(0.065)	0.295	(0.055)	0.295	(0.055)	ns
**TS (%)**										
	0–2	0.130	(0.010)	0.210	(0.010)	0.190	(0.010)	0.190	(0.010)	ns
	2–4	0.055	(0.005)	0.270	(0.020)	0.490	(0.030)	0.490	(0.030)	**
	4–6	0.235	(0.025)	0.180	(0.010)	0.100	(0.010)	0.100	(0.010)	*
	6–8	0.110	(0.010)	0.135	(0.005)	0.495	(0.015)	0.495	(0.015)	***
	8–10	0.220	(0.010)	0.180	(0.020)	1.025	(0.055)	1.025	(0.055)	**
**TC (%)**										
	0–2	0.960	(0.020)	1.260	(0.060)	1.180	(0.030)	1.180	(0.030)	ns
	2–4	0.975	(0.015)	1.080	(0.040)	1.300	(0.040)	1.300	(0.040)	***
	4–6	1.305	(0.015)	0.720	(0.390)	0.875	(0.065)	0.875	(0.065)	ns
	6–8	1.130	(0.020)	1.290	(0.080)	0.800	(0.070)	0.800	(0.070)	*
	8–10	0.600	(0.050)	0.975	(0.015)	0.865	(0.045)	0.865	(0.045)	*
**TP (%)**										
	0–2	0.0120	(0.0010)	0.0155	(0.0005)	0.0205	(0.0015)	0.0205	(0.0015)	*
	2–4	0.0125	(0.0015)	0.0190	(0.0010)	0.0175	(0.0005)	0.0175	(0.0005)	ns
	4–6	0.0135	(0.0015)	0.0170	(0.0001)	0.0155	(0.0010)	0.0155	(0.0005)	ns
	6–8	0.0160	(0.0010)	0.0170	(0.0010)	0.0230	(0.0040)	0.0230	(0.0040)	ns
	8–10	0.0195	(0.0015)	0.0190	(0.0001)	0.0120	(0.0010)	0.0120	(0.0010)	*
**Fe (mg/kg)**										
	0–2	16.5	(0.3)	22.5	(1.1)	30.6	(1.1)	30.6	(1.1)	**
	2–4	17.8	(0.1)	22.3	(0.1)	13.1	(0.5)	13.1	(0.5)	***
	4–6	27.6	(0.4)	16.3	(0.5)	12.7	(0.7)	12.7	(0.7)	**
	6–8	29.0	(0.5)	37.0	(0.2)	24.7	(0.5)	24.7	(0.5)	***
	8–10	15.4	(0.2)	24.5	(2.0)	19.6	(0.1)	19.6	(0.1)	*

Means values ± standard errors and *p*-level of analysis of variance (ANOVA) are indicated (*, *p* < 0.05; **, *p* < 0.01; ***, *p* < 0.001; ns, not significant). Notes: TOC = Total Organic Matter, TN = Total Nitrogen, TS = Total Sulphur, TC = Total Carbon, TP = Total Phosphorus, Fe = Iron; se = standard error.

**Table 2:** Sediments’ chemical composition for each deep layer in each time under anoxic conditions.

Content	Depth	T_0_		T_1_		T_2_		T_3_		
**TOC (%)**	cm	Anoxy Condition		Anoxy Condition		Anoxy Condition		Anoxy Condition		*p*-level
		mean	(se)	mean	(se)	mean	(se)	mean	(se)	
	0–2	0.580	(0.030)	0.970	(0.010)	1.080	(0.010)	1.080	(0.010)	***
	2–4	0.710	(0.010)	1.185	(0.045)	0.530	(0.020)	0.530	(0.020)	***
	4–6	1.000	(0.010)	0.595	(0.055)	1.065	(0.035)	1.065	(0.035)	***
	6–8	0.615	(0.065)	0.055	(0.005)	1.145	(0.025)	1.145	(0.025)	***
	8–10	0.405	(0.015)	0.355	(0.245)	0.255	(0.045)	0.255	(0.045)	ns
**TN (%)**										
	0–2	0.040	(0.010)	0.595	(0.075)	0.435	(0.115)	0.435	(0.115)	ns
	2–4	0.215	(0.065)	0.560	(0.070)	0.610	(0.060)	0.610	(0.060)	***
	4–6	0.220	(0.030)	0.240	(0.020)	0.250	(0.020)	0.250	(0.020)	ns
	6–8	0.100	(0.050)	0.035	(0.015)	0.070	(0.020)	0.070	(0.0200)	ns
	8–10	0.080	(0.010)	0.025	(0.015)	0.045	(0.015)	0.045	(0.015)	ns
**TS (%)**										
	0–2	0.130	(0.010)	0.085	(0.025)	1.030	(0.030)	1.030	(0.030)	***
	2–4	0.055	(0.005)	0.185	(0.025)	0.695	(0.095)	0.695	(0.095)	**
	4–6	0.235	(0.025)	0.205	(0.035)	0.400	(0.020)	0.400	(0.020)	*
	6–8	0.110	(0.010)	0.370	(0.060)	0.175	(0.035)	0.175	(0.035)	**
	8–10	0.220	(0.010)	0.530	(0.110)	0.095	(0.015)	0.095	(0.015)	**
**TC (%)**										
	0–2	0.960	(0.020)	1.185	(0.165)	1.415	(0.255)	1.415	(0.255)	ns
	2–4	0.975	(0.015)	1.160	(0.070)	1.070	(0.040)	1.070	(0.040)	ns
	4–6	1.305	(0.015)	0.720	(0.070)	1.370	(0.050)	1.370	(0.050)	*
	6–8	1.130	(0.020)	0.720	(0.080)	1.600	(0.045)	1.600	(0.045)	**
	8–10	0.600	(0.050)	0.515	(0.015)	2.200	(0.055)	2.200	(0.055)	***
**TP (%)**										
	0–2	0.0120	(0.0010)	0.0235	(0.0015)	0.0225	(0.0015)	0.0225	(0.0015)	*
	2–4	0.0125	(0.0015)	0.0205	(0.0015)	0.0230	(0.0010)	0.0230	(0.0010)	*
	4–6	0.0135	(0.0015)	0.0135	(0.0015)	0.0215	(0.0005)	0.0215	(0.0005)	**
	6–8	0.0160	(0.0010)	0.0125	(0.0015)	0.0195	(0.0005)	0.0195	(0.0005)	ns
	8–10	0.0195	(0.0015)	0.0125	(0.0015)	0.0135	(0.0025)	0.0135	(0.0025)	ns
**Fe (mg/kg)**										
	0–2	16.5	(0.3)	35.0	(2.6)	34.3	(1.1)	34.3	(1.1)	*
	2–4	17.8	(0.1)	28.8	(1.9)	32.0	(0.5)	32.0	(0.5)	**
	4–6	27.6	(0.4)	31.6	(2.2)	27.9	(1.4)	27.9	(1.4)	ns
	6–8	29.0	(0.5)	16.3	(0.6)	25.8	(1.5)	25.8	(1.5)	**
	8–10	15.4	(0.2)	13.1	(0.4)	28.1	(1.9)	28.1	(1.9)	**

Means values ± standard errors and *p*-level of ANOVA are indicated (*, *p* < 0.05; **, *p* < 0.01; ***, *p* < 0.001; ns, not significant). Notes: TOC= Total Organic Carbon, TN= Total Nitrogen, TS= Total Sulphur, TC= Total Carbon, TP= Total Phosphorus, Fe= Iron, se = standard error.

**Table 3:** Carbohydrates, proteins and lipids composition for each deep layer in each time under oxygenic conditions.

Content	Depth	T_0_		T_1_		T_2_		T_3_		
**CHO mg/g**	cm	Oxy Condition		Oxy Condition		Oxy Condition		Oxy Condition		*p*-level
		mean	(se)	mean	(se)	mean	(se)	mean	(se)	
	0–2	0.0071	(0.0006)	0.0068	(0.0002)	0.0070	(0.0020)	0.0070	(0.0020)	ns
	2–4	0.0166	(0.0004)	0.0314	(0.0007)	0.0126	(0.0050)	0.0126	(0.0050)	*
	4–6	0.0051	(0.0003)	0.0123	(0.0020)	0.0010	(0.0000)	0.0010	(0.0000)	*
	6–8	0.0010	(0.0000)	0.0010	(0.0000)	0.0010	(0.0000)	0.0010	(0.0000)	***
	8–10	0.0010	(0.0000)	0.0010	(0.0000)	0.0010	(0.0000)	0.0010	(0.0000)	***
**PRT mg/g**										
	0–2	0.0183	(0.004)	0.0278	(0.0140)	0.0497	(0.011)	0.0814	(0.017)	ns
	2–4	0.0080	(0.003)	0.0322	(0.0160)	0.0420	(0.010)	0.0688	(0.016)	ns
	4–6	0.0267	(0.009)	0.0156	(0.0002)	0.0380	(0.009)	0.0621	(0.015)	ns
	6–8	0.0229	(0.007)	0.0355	(0.0003)	0.0599	(0.020)	0.0347	(0.021)	ns
	8–10	0.0126	(0.005)	0.0234	(0.0015)	0.0301	(0.010)	0.0492	(0.017)	ns
**LIP mg/g**										
	0–2	0.0023	(0.0009)	0.0016	(0.0001)	0.0077	(0.0010)	0.0103	(0.0030)	ns
	2–4	0.0056	(0.0027)	0.0014	(0.0001)	0.0033	(0.0002)	0.0044	(0.0010)	ns
	4–6	0.0018	(0.0008)	0.0010	(0.0004)	0.0032	(0.0001)	0.0042	(0.0010)	*
	6–8	0.0020	(0.0009)	0.0009	(0.0004)	0.0062	(0.0004)	0.0082	(0.0020)	*
	8–10	0.0040	(0.0021)	0.0011	(0.0005)	0.0049	(0.0004)	0.0065	(0.0020)	ns

Means values ± standard errors and *p*-level of ANOVA are indicated (*, *p* < 0.05; **, *p* < 0.01; ***, *p* < 0.001; ns, not significant). Notes: CHO = Carbohydrates, PRT = proteins, LP = lipids, se = standard error.

**Table 4:** Carbohydrates, proteins and lipids composition for each deep layer in each time under anoxic conditions.

Content	Depth	T_0_		T_1_		T_2_		T_3_		
**CHO mg/g**	cm	Anoxy Condition		Anoxy Condition		Anoxy Condition		Anoxy Condition		*p*-level
		mean	(se)	mean	(se)	mean	(se)	mean	(se)	
	0–2	0.0071	(0.0006)	0.0316	(0.0010)	0.0429	(0.0003)	0.0429	(0.0003)	***
	2–4	0.0166	(0.0004)	0.0258	(0.0030)	0.0292	(0.0003)	0.0292	(0.0003)	*
	4–6	0.0051	(0.0003)	0.0062	(0.0006)	0.0052	(0.0040)	0.0052	(0.0040)	ns
	6–8	0.0010	(0.0000)	0.0010	(0.0000)	0.0010	(0.0000)	0.0010	(0.0000)	***
	8–10	0.0010	(0.0000)	0.0010	(0.0000)	0.0010	(0.0000)	0.0010	(0.0000)	***
**PRT mg/g**										
	0–2	0.0183	(0.0040)	0.0558	(0.0170)	0.1201	(0.0180)	0.1844	(0.0180)	**
	2–4	0.0080	(0.0030)	0.0490	(0.0140)	0.1133	(0.0140)	0.1776	(0.0140)	**
	4–6	0.0267	(0.0090)	0.0320	(0.0080)	0.0963	(0.0080)	0.1606	(0.0080)	**
	6–8	0.0229	(0.0070)	0.0289	(0.0080)	0.0932	(0.0080)	0.1575	(0.0080)	**
	8–10	0.0126	(0.0050)	0.0294	(0.0070)	0.0937	(0.0070)	0.1580	(0.0070)	**
**LIP mg/g**										
	0–2	0.0023	(0.0009)	0.0104	(0.0040)	0.0091	(0.0030)	0.0078	(0.0030)	ns
	2–4	0.0056	(0.0027)	0.0049	(0.0020)	0.0036	(0.0020)	0.0029	(0.0020)	ns
	4–6	0.0018	(0.0008)	0.0058	(0.0030)	0.0045	(0.0030)	0.0039	(0.0020)	ns
	6–8	0.0020	(0.0009)	0.0028	(0.0010)	0.0020	(0.0010)	0.0014	(0.0003)	ns
	8–10	0.0040	(0.0021)	0.0023	(0.0010)	0.0016	(0.0006)	0.0010	(0.0001)	ns

Means values ± standard errors and *p*-level of ANOVA are indicated (*, *p* < 0.05; **, *p* < 0.01; ***, *p* < 0.001; ns, not significant). Notes: CHO = Carbohydrates, PRT = proteins, LP = lipids, se = standard error.

**Table 5:** (**a**) Eigen vectors (coefficients in the linear combinations of variables making up principal components) related to the Figure 4a. (**b**) Eigen vectors (coefficients in the linear combinations of variables making up PC’s) related to Figure 4b.

Variable	PC1	PC2	PC3
(**a**)			
**TOC**	−0.201	−0.200	−0.476
**TN**	−0.041	0.392	−0.024
**TS**	0.193	−0.191	0.133
**TC**	0.084	0.036	−0.539
**TP**	0.332	0.021	0.054
**Fe**	0.175	0.307	−0.047
**CHO**	−0.137	−0.062	−0.524
**Chl-a**	0.282	−0.324	−0.214
**PHAE**	0.250	−0.237	−0.163
**PRT**	0.547	0.166	0.005
**LIP**	0.091	−0.490	0.146
**OM**	−0.059	0.335	0.031
**LOM**	−0.076	−0.352	0.287
**BPC**	0.544	0.071	−0.092
(**b**)			
**TOC**	−0.253	−0.080	0.383
**TN**	−0.357	−0.237	0.029
**TS**	−0.220	−0.009	−0.493
**TC**	−0.142	0.422	0.412
**TP**	−0.352	0.028	−0.027
**Fe**	−0.317	0.114	0.270
**CHO**	−0.318	−0.307	0.016
**Chl-a**	−0.251	−0.115	−0.092
**PHAE**	−0.293	−0.070	−0.128
**PRT**	−0.277	0.420	−0.106
**LIP**	−0.126	−0.462	−0.155
**OM**	−0.234	−0.221	0.402
**LOM**	−0.213	0.383	−0.119
**BPC**	−0.271	0.219	−0.359
